# Kaurane-Type Diterpenoids as Potential Inhibitors of Dihydrofolate Reductase-Thymidylate Synthase in New World *Leishmania* Species

**DOI:** 10.3390/antibiotics12040663

**Published:** 2023-03-28

**Authors:** Chonny Herrera-Acevedo, Renata Priscila Barros de Menezes, Natália Ferreira de Sousa, Luciana Scotti, Marcus Tullius Scotti, Ericsson Coy-Barrera

**Affiliations:** 1 Post-Graduate Program in Natural and Synthetic Bioactive Products, Federal University of Paraíba, João Pessoa 58051-900, PB, Brazilmtscotti@gmail.com (M.T.S.); 2 Department of Chemical Engineering, Universidad ECCI, Bogotá, Distrito Capital 111311, Colombia; 3 Bioorganic Chemistry Laboratory, Facultad de Ciencias Básicas y Aplicadas, Universidad Militar Nueva Granada, Cajicá 250247, Colombia

**Keywords:** kauranes, *Leishmania*, Asteraceae, machine learning, DHFR-TS, diterpenes, natural products

## Abstract

The bifunctional enzyme Dihydrofolate reductase-thymidylate synthase (DHFR-TS) plays a crucial role in the survival of the *Leishmania* parasite, as folates are essential cofactors for purine and pyrimidine nucleotide biosynthesis. However, DHFR inhibitors are largely ineffective in controlling trypanosomatid infections, largely due to the presence of Pteridine reductase 1 (PTR1). Therefore, the search for structures with dual inhibitory activity against PTR1/DHFR-TS is crucial in the development of new anti-*Leishmania* chemotherapies. In this research, using the *Leishmania major* DHFR-TS recombinant protein, enzymatic inhibitory assays were performed on four kauranes and two derivatives that had been previously tested against *Lm*PTR1. The structure **302** (6.3 µM) and its derivative **302a** (4.5 µM) showed the lowest IC_50_ values among the evaluated molecules. To evaluate the mechanism of action of these structures, molecular docking calculations and molecular dynamics simulations were performed using a DHFR-TS hybrid model. Results showed that hydrogen bond interactions are critical for the inhibitory activity against *Lm*DHFR-TS, as well as the presence of the *p*-hydroxyl group of the phenylpropanoid moiety of **302a**. Finally, additional computational studies were performed on DHFR-TS structures from Leishmania species that cause cutaneous and mucocutaneous leishmaniasis in the New World *(L. braziliensis, L. panamensis*, and *L. amazonensis*) to explore the targeting potential of these kauranes in these species. It was demonstrated that structures **302** and **302a** are multi-*Leishmania* species compounds with dual DHFR-TS/PTR1 inhibitory activity.

## 1. Introduction

Leishmaniasis is a neglected tropical disease (NTD) caused by *Leishmania* parasites, a type of trypanosomatid protozoa [1]. The disease affects 15 million people globally, presenting in three forms: cutaneous (CL), mucocutaneous (ML), and visceral (VL) [2,3]. Despite public health concerns and the need for control, current treatments, including pentavalent antimony salts as first-line drugs or amphotericin B, pentamidine, miltefosine, or paromomycin as second-line drugs, are frequently toxic, as well as expensive with increasing resistance outbreak [3,4,5]. Liposomal amphotericin B is one of the best treatment options for leishmaniasis, but its effectiveness depends on the patient’s immune status, clinical presentation, and location. In addition, its use is limited in developing countries due to its high cost, side effects, and need for injection [6]. Despite attempts to discover more effective and safe alternatives through drug discovery [1,2,7], limited progress has been made, making the search for new antileishmanial chemotherapies necessary [8].

A metabolic pathway that is traditionally considered a crucial target against trypanosomatid parasites involves the inhibition of dihydrofolate reductase (DHFR) in the biosynthesis of folate-like cofactors [9]. DHFR (EC 1.5.1.3) catalyzes the NADPH-dependent reduction of 7,8-dihydrofolates (H2Fs) to 5,6,7,8-tetrahydrofolates (H4Fs) [10], which are necessary for maintaining adequate intracellular folate concentrations [9,10]. In trypanosomatids, a single, fused gene encodes a bifunctional enzyme that has both the DHFR domain and the thymidylate synthase (TS) domain [11]. This bifunctional enzyme is crucial for the parasite’s survival because folates are essential cofactors for the biosynthesis of purine and pyrimidine nucleotides. As a result, inhibition of this single polypeptide can affect two steps of this essential pathway [12]. In contrast, humans have separate monofunctional polypeptides for DHFR and TS, leading to structural differences and unique roles in human folate production [9]. This makes the DHFR-TS combination an attractive molecular target for the development of antimicrobial agents. In fact, antifolate-based antimicrobial drugs such as methotrexate (MTX), trimethoprim, and pyrimethamine are already in use [9,12].

However, *Leishmania* parasites are auxotrophic for folate, meaning they have a sophisticated metabolic pathway for acquiring folate from the host and incorporating it into intermediate or alternative metabolisms through the action of pteridine reductase (PTR1) [13]. PTR1 (EC 1.5.1.33) transforms conjugated and nonconjugated pterins, including the reduction of biopterin to dihydrobiopterin, and then to tetrahydrobiopterin. This catalytic role is crucial for maintaining vital intracellular levels of tetrahydropterin and has been shown to be an essential component of growth in vivo through gene expression studies [14]. Since PTR1 is less sensitive to the effect of MTX and catalyzes folate reduction, this explains the therapeutic failures of antifolate drugs against trypanosomatid parasites [13,15,16]. Thus, an appropriate strategy would involve searching for dual inhibitors of PTR1 and DHFR as antileishmanial agents [17], and natural compounds are still considered a vast source of bioactive agents [2].

Schmidt et al. conducted a virtual screening of 118 sesquiterpene lactones to evaluate natural products as dual PTR1/DHFR-TS inhibitors against *Trypanosoma brucei*. Among the 29 virtual hits identified, in vitro assays were performed on 10 selected molecules using recombinant PTR1 and DHFR proteins. Five compounds showed an inhibition of over 50% against *Tb*PTR1, while three compounds exhibited inhibition against *Tb*DHFR, with cynaropicrin being the most interesting hit, inhibiting both *Tb*PTR1 and *Tb*DHFR. [18] Specifically, against *Leishmania*, Teixeira et al. evaluated 2,4 diaminopyrimidine derivatives as dual and selective inhibitors of pteridine reductase DHFR/PTR1 from *Leishmania chagasi*. Quinazoline was identified as a selective inhibitor of *Lc*PTR1, and 2,4 diaminopyrimidine derivatives substituted at position 6 were found to be competitive inhibitors of DHFR/PTR1 [19]. Recently, quinoline-linked isatin derivatives showed promising in vitro activity against the promastigote and amastigote form, with 5-bromoindoline, 5-flouroindoline, and 5-trifluoromethoxy indoline derivatives exhibiting significant activity. Folic and folinic acids were able to reverse the antileishmanial effect of these three compounds, confirming their antifolate mechanism via inhibition of DHFR-TS and PTR1 [20].

A class of bioactive naturally-occurring compounds known as kaurane-type diterpenes has been shown to exhibit antileishmanial activity at various levels [21,22,23]. Based on this evidence, a previous in silico and in vitro study was performed on a custom-made library of 360 compounds to select kaurane-type diterpenes against *Leishmania major* PTR1 (*Lm*PTR1). Two kauranes, structures **135** (2β-hydroxy-menth-6-en-5β-yl *ent*-kaurenoate) and **302** (3α-cinnamoyloxy-*ent*-kaur-16-en-19-oic acid), were identified with antileishmanicidal activity against *L. major* through an in silico approach combining machine learning and molecular docking methodologies. The in vitro results verified the accuracy of the classification model. The top-ranked compounds and two semisynthetic derivatives were found to have half-maximal inhibitory concentrations (IC_50_) less than 10 µg/mL, which showed that the inhibitory activity of structure **302** was improved by approximately 60% when a 3-*p*-coumaroyloxy group was used instead of the 3-cinnamoyloxy substituent [24]. These selected compounds can be considered important leads that can be used to obtain more active PTR1 inhibitors. In addition, molecular docking calculations and MD simulations were performed for the entire set of kauranes, and the compounds **302**, and **302a** (3α-*p*-coumaroyloxy-*ent*-kaur-16-en-19-oic acid) were identified as potential multispecies agents against other *Leishmania* species responsible for the clinical diversity of CL and MCL [24]. Based on the previously obtained results, the present study aims to investigate the selection of kauranes that exhibit activity against *L. major* DHFR-TS, along with their potential inhibitory activity in *Leishmania* species of the New World. Furthermore, the study aims to identify potential dual inhibitors of DHFR/PTR1, leveraging the prior findings against PTR1.

## 2. Results and Discussion

### 2.1. Kauranes 302 and Its Derivative 302a Have Dual In Vitro Enzymatic Activity against L. major PTR1/DHFR-TS

**Figure 1:**
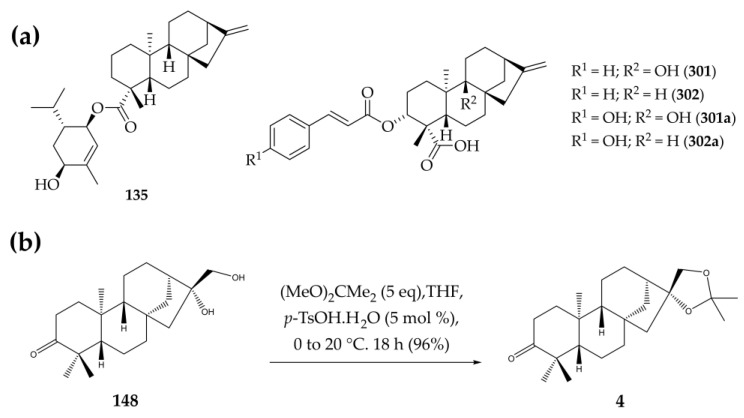
(**a**) Structures of selected kaurane-type diterpenes (**135, 301, 302**) and their derivatives (**301a** and **302a**). (**b**) Synthesis of compound **4** from structure **148**.

The potential dual enzymatic activity of *L. major* PTR1/DHFR-TS for the diterpene esters **135**, **301** (3α-cinnamoyloxy-9β-hydroxy-*ent*-kaur-16-en-19-oic Acid), **302**, **301a** (3α-*p*-coumaroyloxy-9β-hydroxy-*ent*-kaur-16-en-19-oic acid), and **302a** (which have already been evaluated against *L. major* PTR1 [24], as shown in Figure 1a), along with structure **4** (6ß,17-isopropylidenedioxy-*ent*-kauran-3-one), was evaluated using spectrophotometric monitoring of enzymatic activity under a standard DHFR assay. This was performed with a range of test compound concentrations (0.1–128 µM), and methotrexate was used as a positive control. Structure **4** was also selected for the assays, since in previously performed structure-based virtual screening of 360 kaurane-type diterpenes using DHFR-TS of *Leishmania* species of the New World as targets, it was one of the best-ranked molecules, demonstrating selectivity against this target.

The IC_50_ values were calculated based on the concentration–response behavior within the range of 0.1–128 μM, resulting in values ranging from 4.5 to 11.2 μM (pIC_50_ values ranging from 4.95 to 5.35). Then, using the Cheng–Prusoff equation and assuming reversible competitive inhibition and a 1:1 stoichiometry [25], the apparent inhibitory constant (K_i_^app^) was calculated for the selected kauranes using the IC_50_ results, as shown in Table 1.

The evaluated structures showed similar IC_50_ values. Among the six tested diterpenes, structure **135** was the least active, which was contrary to what was observed with PTR1. Structure **301**, which was classified as inactive against PTR1, showed a different behavior against DHFR-TS with a pIC_50_ value above 5.0, and was classified as active against this enzyme, according to the cutoff value used to build the machine learning model of *L. major* (pIC_50_ = −log IC_50_) [24].

Using MolpredictX, a recent web tool developed in the Laboratory of Cheminformatics at the Federal University of Paraíba, which provides predictions for 27 different biological activities, including *L. major*, structures **301** and **301a** were classified as active. This tool provides qualitative predictions of molecule activity (active or inactive) and a quantitative probability of activity based on molecular descriptors [26].

For DHFR-TS, the kaurane-type diterpenes **301, 302**, **301a**, and **302a** showed similar pIC_50_ values above 5.0, indicating that the 9-hydroxyl group at the diterpene moiety is not relevant to the inhibitory activity as observed with PTR1, and suggesting different mechanisms of action for these two enzymes in *Leishmania*. Additionally, the *p*-hydroxyl group has a favorable influence on the inhibitory activity of the evaluated kauranes, reducing the inhibitory constant (K_i_^app^) values by 10–30% for **301a** and **302a**, respectively, compared to kauranes **301** and **302**, which do not have this hydroxyl group present in their structures.

Structures **302** and **302a** showed the lowest K_i_^app^ values among the six tested structures against DHFR-TS (despite both having K_i_^app^ values that are higher than MTX). These two structures also displayed a similar behavior to lower K_i_^app^ values in previous enzymatic assays against *L. major* PTR1 [24], which indicates that these two structures have dual in vitro enzymatic activity against *L. major* PTR1/DHFR-TS, with the 9-hydroxyl group at the diterpene moiety being the critical structural feature for the observed dual action against these targets.

### 2.2. Hybrid Model of L. major DHFR-TS and Molecular Docking Calculations

To examine the mechanism of action of the tested kauranes and determine whether the kauranes that previously showed inhibitory activity against pteridine reductase 1 (PTR1) also act against dihydrofolate reductase-thymidylate synthase (DHFR-TS), a molecular docking study was conducted using a *Lm*DHFR-TS hybrid model built in the YASARA software (YASARA Biosciences GmbH, Vienna, Austria; 2018). The model’s reliability and stereochemical qualities were evaluated through Ramachandran, WHAT IF, and VERIFY 3D plots, as well as Z-scores of dihedrals, which describe the deviation of the model’s quality from the average high-resolution X-ray structure. The Ramachandran plot showed that 96.9% of residues were in the most favored regions, with 99.5% in allowed regions and only 0.5% (corresponding to five amino acids) in the outlier region, indicating that the *Lm*DHFR-TS model was satisfactory (Supplementary Material) [27].

The VERIFY 3D (https://services.mbi.ucla.edu/SAVES/, accessed on 3 January 2023) results showed that 92.6% of residues had an averaged 3D-1D score of ≥0.2, indicating a reliable model. The coarse packing quality control of the *Lm*DHFR-TS model, evaluated using WHAT IF, showed a mean score of −0.594, with only 1.7% of residues (8 of 520 amino acids) scoring −5.0 or lower. The dihedral quality was classified as optimal for the *Lm*DHFR-TS hybrid model, with values above 1.085 [28].

Molecular docking calculations were performed for the selected kaurane dataset and derivatives **301a** and **302a** using Molegro 6.0 software, which employs the MolDock scoring function. The previously validated *L. major* DHFR-TS hybrid model was used, and the results were consistent with those obtained from enzymatic assays. The MolDock scores ranged from −62.85 to −81.43 kJ/mol, with all structures showing higher scores than the positive control MTX (−107.60 kJ/mol). Kaurane **302** (−76.53 kJ/mol) and its derivative **302a** (−81.43 kJ/mol), which exhibited the highest inhibitory activity against *L. major* DHFR-TS in the enzymatic assay, also had the lowest MolDock scores among the seven evaluated kaurane-type diterpenes (Table 2). In addition, molecular docking calculations were performed for 5-benzyl-6-(cyclohexylmethyl) pyrimidine-2,4-diamine, a 2,4-diamine derivative that exhibited similar affinity to *L. chagasi* PTR1 and DHFR-TS (with a *Ki^Lc^*^PTR1^/*Ki^Lc^*^DHFR-TS^ ratio of 0.68). The compound was found to have a similar MolDock score (−72.38 kJ/mol) compared to structure **302** and its derivative **302a,** which showed enzymatic inhibition of both *L. major* PTR1 and DHFR-TS [19].

**Figure 2:**
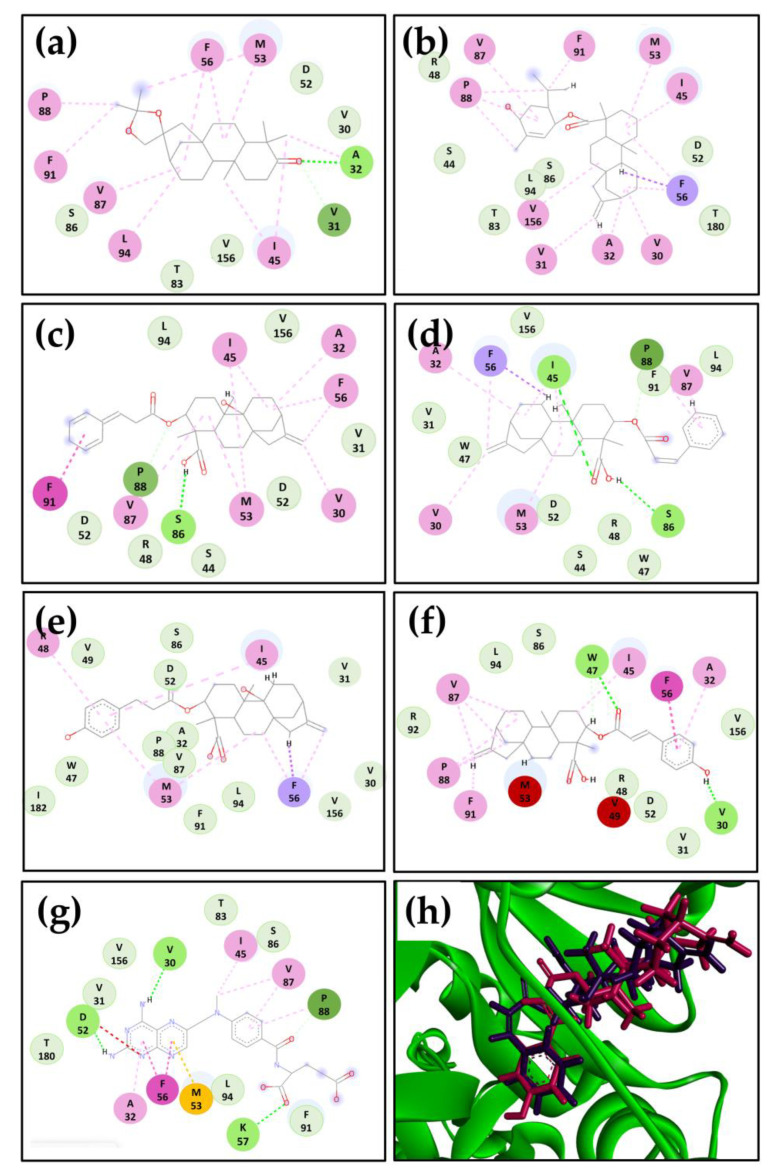
Two-dimensional residual interaction diagrams in the active site of *L. major* DHFR-TS for: (**a**) structure **4**, (**b**) structure **135**, (**c**) structure **301**, (**d**) structure **302**, (**e**) structure **301a**, (**f**) structure **302a** and (**g**) methotrexate (MTX). Interacting residues are shown as colored circles depending on the interactions (as colored dashed lines): H-bond (lime), van der Waals (green), π–σ (purple), π– alkyl (pink), π–π (fuchsia), unfavorable (red), and carbon H-bond (teal) interactions. (**h**) Docking conformations of structure **302** (purple) and its derivative **302a** (pink) in the active site of *L. major* DHFR-TS (green).

Using a two-dimensional analysis, critical interactions with active site amino acid residues of the enzyme were identified. It was observed that hydrogen bond interactions are directly related to the IC_50_ values obtained in the enzymatic assay. Structures **302** and **302a**, which had the lowest IC_50_ values, showed two hydrogen bond interactions involving residues I45 and S86 for 302 and W47 for **302a**, and the carbon-19 of these two kauranes (Figure 2d–f). The interaction with residue S86 was also observed for structure **301**, which had a moderate IC_50_ value among the tested structures, with the only hydrogen bond interaction being observed in this kaurane (Figure 2c). This behavior was also observed in structure **4** (Figure 2a), which only interacted with residue A32 through hydrogen bonds.

The positive control, MTX, showed three hydrogen bond interactions with residues D52, K57, and V30. The interaction with V30 potentially has a crucial role in the inhibition of DHFR-TS. This interaction was only observed in the derivative **302a**, which had the highest inhibitory activity among the tested molecules. Interestingly, this hydrogen bond interaction was formed with the *p*-hydroxyl group of the phenylpropanoid moiety of **302a**, reinforcing the importance of this structural feature in the dual *L. major* PTR1/DHFR-TS inhibitory activity. 

In addition, the analysis of the docking conformations revealed the phenylpropanoid moiety of structures **302** and **302a** adopted a similar conformation in the active site of *L. major* DHFR-TS, with the *p*-hydroxyl group identified as a crucial feature for the observed inhibitory activity (Figure 2h).

Residue F56 also plays a key role in the inhibition of *L. major* DHFR-TS when it interacts with aromatic regions in the kaurane series, as the most active molecules exhibited a π–π interaction between the phenyl group of the amino acid and the pteridine ring and phenylpropanoid moiety of MTX and structure **302a**, respectively. A different behavior was observed for structure **302** and the derivative **301a**, which had intermediate inhibitory activity against DHFR-TS. These two molecules, along with structure **135**, showed a π–σ interaction with the hydrogens of the kaurane region. Structure **302a** was the only structure that showed an unfavorable interaction with residue M53, which is important for MTX, with a π–sulfur interaction being established (Figure 2f,g).

### 2.3. Kaurane 302 and Its Derivative 302a May Have the Potential to Inhibit DHFR-TS in Different Species of Leishmania from the New World

Leishmaniasis contracted in North and South America is referred to as “new world leishmaniasis” [29]. Studying this type of species is crucial for the control and elimination of the disease, as there is a high diversity of *Leishmania* species in the Americas, with high concentrations of different species found in countries such as Brazil and Colombia, leading to a significant disease burden [30]. Some of the main New World *Leishmania* species include *Leishmania panamensis*, which is the primary cause of cutaneous leishmaniasis (CL) in Panama and has been found to infect both anthropophilic vectors and mammalian reservoirs [31]; *Leishmania braziliensis*, a pathogenic agent of CL and mucocutaneous leishmaniasis (MCL), primarily distributed in South and Central America [29,30,31,32]; and *Leishmania amazonensis*, an etiological agent of diffuse CL and tegumentary leishmaniasis (TL) [33]. In previous research, molecular docking calculations and MD simulations using PTR1 hybrid models of *L. braziliensis*, *L. amazonensis*, and *L. panamensis* identified kauranes **135**, **302**, and its derivative **302a** as potential multispecies agents [24].

To evaluate their potential dual inhibitory activity against PTR1 and DHFR-TS, hybrid models of DHFR-TS for these three *Leishmania* species were built, and molecular docking calculations and MD simulations were performed using the four kauranes and two derivatives, which were previously tested against the DHFR-TS recombinant. The Ramachandran plot of these three hybrid models showed that the main possible chain conformations included more than 97.2% of residues in the most favored regions for the three hybrid models, with 99.7% of residues in allowed regions. All models showed three residues (0.3%) in disallowed regions (outliers; Supplementary Material).

Additionally, a multiple sequence alignment of DHFR sequences for *L. major*, *L. braziliensis*, *L. panamensis*, and *L. amazonensis* species, along with the *Homo sapiens* DHFR sequence, was carried out. The results showed that the percentage of similarity between *Homo sapiens* and *Leishmania* species is below 25%, with only five conserved residues out of the main 15 residues associated with the interaction between DHFR with kaurane-type diterpenes. However, among the four Leishmania species used in this study, similarity values close to 80% were obtained.

The analysis of the docking results showed that for *L. braziliensis*, the tested structures had similar VINA score values, except for derivative **302a**, which presented the lowest affinity value (−11.17 kcal/mol). All structures had lower docking values compared to MTX (−9.64 kcal/mol), as seen in Table 3. By analyzing the interactions between the tested kauranes and the flexible residues of the active site of *L. braziliensis* DHFR-TS, it was found that the unsaturation of carbon-17 is crucial for the inhibition of **301**, **302**, and their derivatives **301a** and **302a** with the enzyme, via π-alkyl interactions with Y91 and M53. This interaction was also observed in MTX through a π-sulfur interaction with the thiol group of methionine. In addition, the potential inhibitory activity observed for structure **4** was related to a hydrogen bond interaction between Q48 and the carbonyl group of carbon-3, as well as the presence of a 1,3-dioxolane group. Neither structure **301** nor **302** interacted with the phenylpropanoid moiety of their structures, which is different from what was previously observed with *L. major* DHFR-TS.

**Figure 3:**
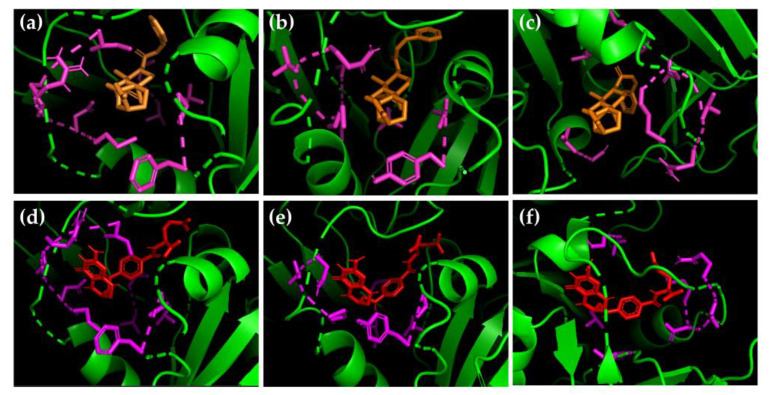
Best pose of structure **302** (orange) and MTX (red) in the active site of (**a**–**d**) *L. amazonensis (***b**–**e**) *L. braziliensis* and (**c**–**f**) *L. panamensis* DHFR-TS (green). Flexible amino acids are marked in pink.

For *L. panamensis*, both derivative structures **301a** and **302a** presented the lowest VINA score values, −12.55 kcal/mol and −12.54 kcal/mol, respectively, showing a higher inhibitory activity compared to the four kauranes and the control, MTX (Table 3). Structures **301** and **302** did not show any π-alkyl interaction with Y91 (Figure 3b), with mainly van der Waals forces observed with flexible residues such as V31, V49, and V156. Kaurane **301** established a hydrogen bond between the carboxylic group of Carbon 4 and residue Q48. This interaction was also observed for MTX, however, a negative–negative unfavorable interaction with D52 affected the affinity value for this compound. A common alkyl interaction between V31 and the unsaturation of carbon-17 of structure **135**, and between V31 and the 1,3-dioxolane group of structure **4**, was also observed.

In the same manner, *L. amazonensis* exhibited a behavior similar to that of *L. braziliensis*, with VINA score values ranging from −10.52 to −11.14 kcal/mol, all of which showed lower affinity values compared to MTX (−9.54 kcal/mol). The latter only showed three interactions with the flexible residues in the active site of the enzyme, including two van der Waals interactions with V49 and Q48 and a π-sulfur interaction between the sulfhydryl group of M53 and the pteridine ring. Structures **301** and **302** displayed the same interactions, which were classified into three groups: π-alkyl with M53 and Y91; alkyl with V87; and van der Waals with V49, V31, and V156. On the other hand, structure **4** was the only kaurane that exhibited a hydrogen bond interaction with Q48, which might explain the slight difference in its affinity value.

Figure 3 displays the complex between the best-docked pose of structure **302**, the potential multispecies dual DHFR-TS/PTR1 inhibitor, and each of the three DHFR-TS hybrid models built in this study. For the *L. braziliensis* and *L. amazonensis* species (Figure 3a,b), similar poses and intermolecular interactions were observed, highlighting the π-alkyl interactions of Y91 and M53 with the double bond of carbon-17.

In contrast, *L. panamensis* showed a different three-dimensional conformation in the active site of DHFR-TS, with a different spatial position for the phenylpropanoid moiety compared to the other two species of *Leishmania*. Additionally, residue Y91, which was a key residue in the interaction of the evaluated structures with the enzyme in *L. amazonensis* and *L. braziliensis* species (Figure 3a,b), did not interact with the unsaturation of carbon-17. This same pattern was also observed for MTX, where Y91 did not appear to be a relevant amino acid for the inhibitory activity.

### 2.4. Molecular Dynamics Simulations for L. major and L. braziliensis DHFR-TS Interacting with 302 and MTX

To validate the hybrid models built for the different *Leishmania* species used in this study and evaluate the protein–ligand stability of structure **302** and its derivative **302a**, molecular dynamics (MD) studies were performed on *L. major* and *L. braziliensis* DHFR-TS using MTX as a reference ligand. Among the three species of *Leishmania* used in this study, *Leishmania braziliensis* was selected to perform MD simulations, because this species is responsible for the most cases in the New World. *L. braziliensis* causes CL and MCL, which are prevalent in Brazil, Peru, Colombia, and other countries in Central and South America. *L. amazonensis* and *L. panamensis* are also causative agents of CL and MCL, but they are less common than *L. braziliensis* [34].

**Figure 4:**
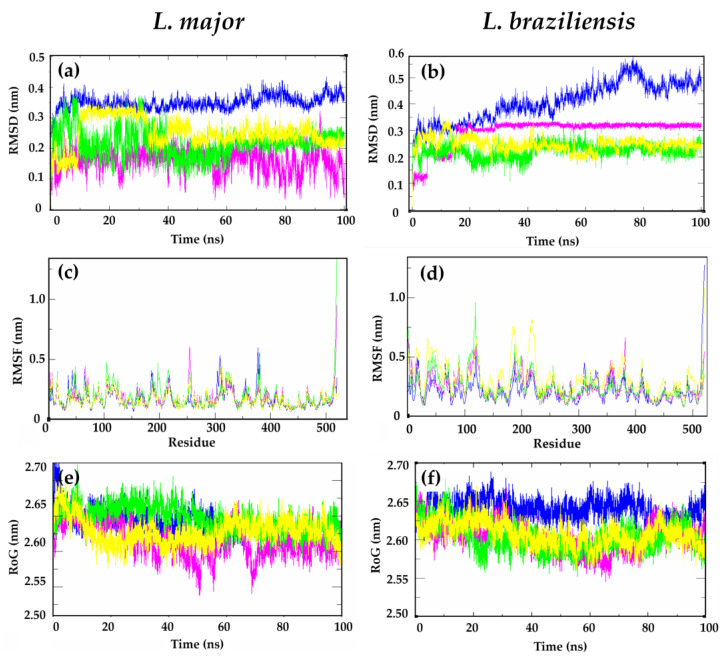
(**a**,**b**) Root-mean-square deviation (RMSD), (**c**,**d**) root-mean-square fluctuation (RMSF), and (**e**,**f**) radius of gyration (RoG) values within the *L. major* DHFR-TS and *L. braziliensis* DHFR-TS binding site, obtained after molecular dynamics simulations. Apoenzyme (blue); DHFR-TS:MTX complex (yellow); DHFR-TS: **302** complex (pink); DHFR-TS: **302a** complex (light green).

Initially, root-mean-square deviation (RMSD) analyses were conducted to assess the structural stability of the receptor frame. These analyses measured the distance between different positions of a set of atoms over time (in nm) [35]. For *L. major* DHFR-TS, during the first 30 ns, similar levels of perturbation were observed, with RMSD values ranging from 0.10 to 0.35 nm for structures **302**, **302a**, MTX, and the apoenzyme (apo*Lm*DHFR-TS, the protein without the ligand). After 30 ns, the protein in complex with structure **302** and its derivative **302a** showed increased stability, with lower RMSD values compared to apo*Lm*DHFR-TS (Figure 4a). Structure **302**a showed a similar pattern to the complex *Lm*DHFR-TS:MTX. In the case of *L. braziliensis*, the apo*Lb*DHFR-TS complex showed a gradual increase in RMSD values from 0.20 to 0.60 nm over the course of the 100 ns simulation. In contrast, the *Lb*DHFR-TS:**302a** and *Lb*DHFR-TS: MTX complexes exhibited more stable structures, with RMSD values ranging from 0.20 to 0.25 nm (Figure 4b). This suggests that structure **302a** enhances the stability of the complex with DHFR-TS in both *Leishmania* species, similar to the stability conferred by MTX. In addition, the *Lb*DHFR-TS:**302** complex maintained a relatively constant RMSD value after 30 ns until the end of the simulation, which was consistently higher than that observed for MTX and **302a**.

Afterward, we analyzed the flexibility of residues with different ligands using root-mean-square fluctuation (RMSF) values. Similar patterns were found in both *L. major* and *L. braziliensis* during the entire dynamic simulations (Figure 4c,d). Regions with defined tertiary structures (α-helices or β-sheets) showed similar RMSF values (0.1 to 0.3 nm) for structure **302** and its derivative **302a** in complex with *L. major* DHFR-TS, as well as for the apoenzyme. However, the control compound MTX presented higher RMSF values, particularly in loop regions of the protein. On analyzing the RMSF values in *L. braziliensis* DHFR-TS, the *Lb*DHFR-TS:**302** and the apoenzyme showed similar behaviors over the simulation time, while structure **302** had higher fluctuations in loop regions than MTX and the uncomplexed protein, especially in the region from A113 to T121, where values ranging from 0.30 nm to 0.63 nm were observed. Despite this, in regions with defined tertiary structure, both MTX and the kaurane **302a** showed RMSF values lower than 0.30 nm, which indicates low flexibility in *L. major* DHFR-TS when complexed (Figure 4d).

In addition, we observed the evolution of the packing level of *L. major* and *L. braziliensis* DHFR-TS through the radius of gyration (RoG) values. For *L. major*, the complexes with structure **302** and its derivative **302a** showed no difference in RoG values compared with the control MTX and apo*Lm*DHFR-TS (ranging from 2.55 nm to 2.70 nm), indicating high stability and low fluctuations in the tertiary structure (Figure 4e).

For *L. braziliensis*, the RoG values for DHFR-TS were different for the evaluated complexes compared to the apo*Lb*DHFR-TS. During the first 30 ns of the simulation, no differences in RoG values were observed (RoG of approximately 2.68 nm). However, after this time, the complexes *Lb*DHFR-TS:**302**, *Lb*DHFR-TS:**302**a and *Lb*DHFR-TS:MTX demonstrated different behaviors, with a reduction in the RoG value (approximately 2.61 nm). This indicates that structure **302** and its derivative **302a** stably folded after the simulation, compared to the apoenzyme, which remained at a constant value during the 100 ns test period (Figure 4f).

### 2.5. Free Energy Calculations by the Molecular Mechanics Poisson–Boltzmann Surface Area Approach (MM/PBSA) Method

After the molecular dynamic simulations were completed, the binding free energies for the complexes of structures **302** and **302a**, as well as MTX with *L. major* DHFR-TS and *L. braziliensis* DHFR-TS, were calculated using the MM/PBSA method. Kaurane **302** and its derivative **302a** in complex with *L. major* DHFR-TS reached similar binding free energy values of −138.2 kJ/mol and −134.2 kJ/mol, respectively, which were both higher than the value measured for the complex *Lm*DHFR-TS: MTX, which was −140.1 kJ/mol. Conversely, the complexes *Lb*DHFR-TS:**302** (−134.8 kJ/mol) and *Lb*DHFR-TS:**302a** (−144.5 kJ/mol) reached a lower binding free energy value compared to the complex *Lb*DHFR-TS: MTX (−95.9 kJ/mol). Nevertheless, for both *Leishmania* species, similar energetic contributions were observed, which were linked to the structural features of the evaluated molecules (Table 4).

For the complexes with 302 and 302a in both *Leishmania* species, the van der Waals, electrostatic, and solvent-accessible surface area (SASA) parameters showed negative contributions to the binding free energy. The van der Waals parameter had the highest negative contribution in *L. braziliensis*, and these results are directly related to the molecular docking calculations, where—mainly in New World *Leishmania* species—this type of interaction is fundamental for the stability of the DHFR-TS-diterpenoid complexes. For *L. major*, the electrostatic parameter contributed negatively to the binding free energies for 302 and MTX; however, its contribution in 302a was close to 50%. In this same way, for structure 302 and its derivative 302a complexed with *L. braziliensis*, electrostatic interactions were significatively minor compared to the contribution observed for MTX, which had a higher contribution to the total binding energy. Finally, for all molecules, polar solvation had a positive contribution to the total energy value, with larger contributions to the complexes with MTX in both evaluated *Leishmania* species.

## 3. Materials and Methods

### 3.1. LmDHFR-TS Enzyme Inhibition Assay

Purification and kinetic characterization of the recombinant *Lm*DHFR-TS protein was performed according to the previously reported procedures [36,37]. The in vitro evaluation of selected diterpenoids (i.e., **4**, **135, 301**, **302**, **301a**, and **302a**) for inhibitory activity against *Lm*DHFR-TS was conducted using a spectrophotometric assay under standard DHFR conditions. The assay consisted of *Lm*DHFR (2.7 nM), bovine serum albumin (BSA, 1 mg/mL), *N*-[tris(hydroxymethyl)-methyl]-2-aminoethanesulfonic acid (TES) buffer (100 mM, pH 7.0, 150 mM β-mercaptoethanol, 2 mM ethylenediaminetetraacetic acid (EDTA)), and nicotinamide adenine dinucleotide phosphate (NADPH, 100 μM) with varying concentrations of the test compounds (0.1–128 μM). The reaction was initiated by adding the substrate (7,8-dihydrofolate (H2F), 20 μM) and was monitored for 360 s at 340 nm (i.e., oxidation of NADPH to NADP+) to determine the initial reaction rate (Vo) through linear regression analysis of the resulting absorbance profile. All measurements were performed in triplicate and MTX was used as a positive control. The resulting Vo values were used to calculate the % inhibition, as 100 − (Ri/Rc × 100), where Ri is the Vo in the presence of the inhibitor and Rc is the Vo in the absence of inhibitors (1% DMSO *v*/*v* final concentration). The % inhibition was measured for at least five concentrations (0.1–128 μM) for each test compound (diterpenoids and MTX), and concentration–response curves (% inhibition vs. Log [inhibitor]) were constructed using nonlinear regression to determine the IC_50_ using GraphPad Prism 7.0 (GraphPad, San Diego, CA, USA). The K_i_^app^ values were finally calculated using the Cheng–Prusoff equation for competitive inhibition with a 1:1 stoichiometry and reversible inhibitor-binding reactions: K_i_^app^ = IC_50_/(1 + [S]/K_m_), where [S] is the substrate (H2F) concentration and K_m_ is the Michaelis constant. The substrate K_m_ was calculated during the kinetic characterization of the purified, recombinant *Lm*DHFR-TS and was determined to be 2.4 ± 0.7 μM.

### 3.2. Isolation of Compound 148

Kaurane-type diterpene 148 was isolated from *Euphorbia graminea* Jacq. (Euphorbiaceae), which was propagated under greenhouse conditions from commercially available seeds (Swallowtail Garden Seeds, Santa Rosa, CA, USA). The aerial part (128 g) of two-month-old plants of *E. graminea* was extracted with 96% ethanol, and the raw extract (11.2 g) was purified by column chromatography (CC) using a gradient elution of n-hexane to methanol, yielding fifteen fractions. The purification of fraction 7 was then performed independently by flash column chromatography on SiO_2_ using a mobile phase of a 7:3 mixture of n-hexane and ethyl acetate, which resulted in the isolation of diterpene 148 (35.6 mg). Its spectroscopic data, including NMR and HRMS, were found to match those of the previously isolated compound *ent*-kaurane-3-oxo-16α,17-diol [38].

### 3.3. Synthesis of 16ß,17-Isopropylidenedioxy-ent-kauran-3-one (4)

Compound 4 was synthesized from 148 using a previously reported procedure [39]. Briefly, compound 148 (24 mg, 0.075 mmol) and tetrahydrofuran (THF) (4 mL) were mixed in a 10 mL round-bottom flask by stirring at 0 °C. Then, 2,2-dimethoxypropane (46 µL, 0.375 mmol) and *p*-toluenesulfonic acid monohydrate (0.75 mg, 0.375 µmol) were added. The reaction mixture was stirred at 0 °C for 2 h, allowed to warm to 20 °C, and then stirred at this temperature for 16 h. The reaction was then quenched with saturated NaHCO_3_ (3 mL) and extracted with CH_2_Cl_2_ (3 × 3 mL). The CH_2_Cl_2_ extract was separated, washed with 10% NaCl (2 × 3 mL), dried over MgSO_4_, filtered, and concentrated under reduced pressure to obtain the structure 4 (26 mg, 96%); [α]_D_^20^ –41.8 (c 0.04, CHCI_3_); ^1^H NMR (400 MHz, CDCl_3_) δ_H_ 4.22 (d, J = 8.3 Hz, 1H), 3.61 (d, J = 10.5 Hz, 1H), 2.41 (dd, J = 8.1, 6.3 Hz, 2H), 2.33–2.28 (m, 1H), 1.85 (dd, J = 10.4, 3.6 Hz, 1H), 1.76–1.72 (m, 1H), 1.68–1.65 (m, 2H), 1.64–1.61 (m, 1H), 1.52–1.47 (m, 1H), 1.47–1.44 (m, 1H), 1.37 (s, 3H), 1.33 (s, 3H), 1.27–1.25 (m, 3H), 1.24–1.23 (m, 1H), 1.23–1.19 (m, 3H), 1.14–1.11 (m, 1H), 0.99 (s, 3H), 0.91 (s, 3H), 0.88 (s, 3H), 0.82 (d, J = 8.4 Hz, 1H)*;* ^13^C NMR (100 MHz, CHCI_3_*)* δ_C_ 217.6, 193.0, 109.3, 79.6, 69.5, 55.2, 54.7, 52.3, 47.4, 44.1, 40.6, 40.4, 37.8, 37.3, 37.2, 34.5, 27.6, 27.4, 27.2, 26.7, 20.3, 19.4, 17.8; HREIMS [M+H]^+^ *m/z* 361.2724 (calcd. for C_23_H_37_O_3_, 361.2743).

### 3.4. Hybrid Models of Leishmania DHFR-TS

Hybrid models of the dihydrofolate reductase-thymidylate synthase (DHFR-TS) of different *Leishmania* species were constructed using YASARA software (YASARA 18.4.24, Vienna, Austria: YASARA Biosciences GmbH, 2018) [40]. The FASTA sequences of *L. major* DHFR-TS (P07382), *L. braziliensis* DHFR-TS (A4H4P8), *L. panamensis* DHFR-TS (S5M3K7), and *L. amazonensis* DHFR-TS (P16126) were obtained from the UNIPROT database (https://www.uniprot.org/, accessed on 28 December 2022). The constructed hybrid models were validated through stereochemical quality assessment using PROCHECK [41]. PROCHECK evaluated molecular diversity through several stereochemical parameters, including the torsional angles of the main chain, side chain torsional angles, bad contacts or steric impediments, and planarity. PROCHECK generated a Ramachandran graph [27], which verified the allowed and prohibited regions of the main amino acid chain. The structural quality was evaluated using VERIFY 3D software (https://services.mbi.ucla.edu/SAVES/, accessed on 3 January 2023) and WHAT IF (https://swift.cmbi.ru.nl/servers/html/index.html, accessed on 5 January 2023). VERIFY 3D software analyzes the compatibility of the protein sequence with its 3D structure based on the chemical environment, while WHAT IF analyzes various structural parameters, such as atomic contacts between residues. The Discovery Studio Visualizer (BIOVIA, Dassault Systèmes, Discovery Studio Visualizer, v21.1.0.20298, San Diego: Dassault Systèmes, 2020) was used to visualize the modeled protein [24].

### 3.5. Molecular Docking Calculations

The hybrid model of *L. major* DHFR-TS in complex with methotrexate (PDB ID: MTX) was used for the molecular docking calculations of the six kaurane-type diterpenes using Molegro 6.0.1 software. All water molecules were removed from the enzyme structures and both the enzyme and compound structures were prepared with the same default parameters in the same software package. MolDock was used as the score function, and the internal ES, internal H-bond, and Sp2-Sp2 torsions were all checked as the ligand evaluation criteria. The molecular docking procedure was run 10 times, using the MolDock SE algorithm, with a maximum of 1500 interactions, a maximum population size of 50, a maximum of 300 steps, a neighbor distance factor of 1.00, and a maximum of 5 poses returned. A grid with a 15 Å radius and 0.30 Å resolution was used to cover the ligand-binding site for the enzyme structure [42,43].

For *L. braziliensis*, *L. panamensis*, and *L. amazonensis* DHFR-TS, the docking calculations were performed using the Autodock/Vina (1.1.2) plug-in for PyMOL (1.3r2) under a Python 2.5.2 environment for Windows. The minimized structure was located in a cube with dimensions of 22.5 Å × 22.5 Å × 22.5 Å and a grid spacing of 0.375 Å at the geometric center of the binding pocket (coordinates for *L. braziliensis*: 43.01, 23.70, 1.67; *L. panamensis*: 43.64, 24.00, 1.50; and *L. amazonensis*: 43.49, 24.96, 1.95), which was identified through cavities analysis in Molegro 6.0.1. Flexible residues in the binding site were selected for each model: *L. braziliensis* I30, V31, Q48, V49, M53, V87, and V156; *L. amazonensis*: Q30, V31, Q48, V49, M53, V87, and V156; *L. panamensis*: I30, V31, Q48, V49, D52, M53, S86, and V87. The docking poses were classified based on their docking scores, such as the free energy or affinity, and each calculation was performed in three replicates. Methotrexate (MTX) was used as a control. The two-dimensional residual interaction diagrams were visualized on the Discovery Studio Visualizer (BIOVIA, Dassault Systèmes, Discovery Studio Visualizer, v21.1.0.20298, San Diego: Dassault Systèmes, 2020) [24].

### 3.6. Molecular Dynamics Simulations

Molecular dynamics simulations were carried out using Gromacs 5.0.5 on an Ubuntu 12.04 server [44,45]. Structure 302, its derivative 302a, MTX, and the hybrid models of *L. major* and *L. braziliensis* DHFR-TS were used as inputs for the simulations [40]. *Leishmania braziliensis* was selected to perform MD simulations, because this species is responsible for the most cases in Central and South America. Kaurane-type diterpene 302 and its derivative 302a were selected for MD simulations because they were previously found to have half-maximal inhibitory concentrations (IC_50_) of less than 10 µg/mL against PTR1. It was shown that the inhibitory activity of structure 302 improved by approximately 60% when a 3-*p*-coumaroyloxy group was used instead of the 3-cinnamoyloxy substituent. Additionally, these two structures showed the lowest K_i_^app^ values among the six tested structures against DHFR-TS. These two structures also displayed a similar behavior with lower K_i_^app^ values in previous enzymatic assays against *L. major* PTR1, indicating their potential as dual PTR1/DHFR inhibitors.

For the tested structures, hydrogen atoms and corresponding charges for the ligands were added using the AM1-BCC charge scheme in UCSF Chimera, and the ligand topologies were generated automatically with the ACPYPE script. The protein topologies were obtained in Gromacs using the Amber 99SB force field and the TIP3P water model. Solvation was performed in a triclinic box with a margin distance of 1.0 nm and 0.1 M NaCl was added to the complexes and proteins by randomly replacing water molecules until neutrality was achieved [35,43,44,45]. The systems were energy-minimized for 2000 steps using the steepest descent method. Then, NVT equilibration was performed at 310 K for 50 ps followed by NPT equilibration for 500 ps, using the Parrinello–Rahman method at 1 bar with position restraints. The solute position restraints were then released, and a production run was performed for 5 ns while maintaining constant temperature and pressure at 310 K and 1 bar, respectively. The coordinates were recorded in a 1 fs time step, and electrostatic forces were calculated using the particle-mesh Ewald method. All simulations used periodic boundary conditions, and covalent bond lengths were constrained by the LINCS algorithm.

### 3.7. Binding Free Energies Using the Molecular Mechanics Poisson–Boltzmann Surface Area (MM/PBSA) Method

The binding free energies were calculated using the molecular mechanics Poisson–Boltzmann surface area (MM/PBSA) method based on the trajectories obtained from the molecular dynamics simulations [43,44,45]. The calculation of free binding energy of the protein-binding complex in the study of the molecular behavior of enzymes and their respective ligands was evaluated using the molecular mechanics Poisson–Boltzmann surface area approach (MM/PBSA) method [46]. The GROMACS g_mmpbsa module [47,48] was applied to estimate the bond-free energy of the selected complex using the trajectory files obtained in the molecular dynamics simulation. The GROMACS MM-PBSA calculation consisted of three steps. First, the potential energy in the vacuum was calculated, and then, the energies of polar and, finally, nonpolar solvation were estimated. The nonpolar solvation energy was calculated using the solvent-accessible surface area model (SASA). The required input files and solvation energy values were then selected to evaluate the following energetic components: van der Waals energy, electrostatic energy, polar energy of solvation, nonpolar solvation energy, and free energy of bonding.

## 4. Conclusions

This study identified compounds **302** (3α-cinnamoyloxy-*ent*-kaur-16-en-19-oic acid) and **302a** as potential inhibitors of both PTR1 and DHFR-TS in *L. major*, building upon previous findings of PTR1 inhibition [20]. Both **302** and **302a** displayed in vitro inhibitory activity against *L. major* DHFR-TS, with IC_50_ values of 6.3 and 4.5 µM, respectively. Additionally, other kaurane-type diterpenes, such as synthesized structure **4**, also inhibited DHFR-TS in vitro with an IC_50_ value of 7.6 µM. Structures **301** and **301a**, which were previously classified as inactive against PTR1, also showed inhibitory activity against DHFR-TS, verifying the results obtained from MolpredictX. Furthermore, molecular docking calculations using a hybrid model of *L. major* DHFR-TS allowed evaluation of the mechanism of action of the tested kauranes. The *p*-hydroxyl group of the phenylpropanoid moiety of structure **302a** was found to play a crucial role in the inhibition of DHFR-TS.

Additionally, hybrid models for three *Leishmania* species with high incidence in Central and South America were constructed. The best docked results for structure **302** and its derivative **302a** in the three hybrid models showed a correlation between the affinity values obtained from the molecular docking and some structural features of the kauranes, such as the presence of an unsaturation at carbon-17 that interacts with the amino acids of DHFR-TS through π-alkyl interactions, making these two structures potential multispecies inhibitors. Furthermore, the molecular dynamics simulation, in addition to validating the hybrid models, confirmed the results previously obtained from the molecular docking calculations. So, this study presents a valuable approach for identifying potential dual PTR1/DHFR-TS inhibitors, contributing to the development of alternative chemotherapy strategies against these diseases.

## Figures and Tables

**Table 1:** Results of enzymatic activity against *L. major* dihydrofolate reductase (*Lm*DHFR) for selected kaurane-type diterpenes.

Compound	4	135	302	301	302a	301a	MTX
IC_50_ (µM)	7.6	11.2	6.3	8.8	4.5	7.9	1.4
Confidence Interval (95%)	6.9–8.1	10.2–12.1	5.8–6.9	8.0–9.9	3.9–5.2	7.1–8.4	1.1–1.8
K_i_^app^	0.81	1.20	0.68	0.94	0.48	0.85	0.15

**Table 2:** MolDock scores of six kaurane-type diterpenes, MTX, and a 2,4-diamine derivative (a dual inhibitor of PTR1/DHFR-TS [19]) against *L. major* DHFR-TS. SD = standard deviation; RMSD values = root-mean-square deviation.

Structure	MolDock Score (kJ/mol)	RMSD (A)	SD
**4**	−70.25	0.68	5.7
**135**	−62.85	1.23	8.6
**301**	−73.34	1.09	10.3
**302**	−76.53	1.13	4.9
**301a**	−72.26	0.89	9.8
**302a**	−81.43	1.29	6.4
2,4-diamine	−72.34	1.55	8.9
MTX	−107.60	0.24	5.9

**Table 3:** The VINA score values for six tested structures and MTX (methotrexate) for dihydrofolate reductase-thymidylate synthase (DHFR-TS) of *Leishmania braziliensis*, *Leishmania panamensis*, and *Leishmania amazonensis*. SD = standard deviation; RMSD values = root-mean-square deviation.

	*L. braziliensis*			*L. panamensis*			*L. amazonensis*		
Structure	VINA Score (kcal/mol)	SD	RMSD	VINA Score (kcal/mol)	SD	RMSD	VINA Score (kcal/mol)	SD	RMSD
**4**	−10.70	0.05	0.13	−10.96	0.07	0.46	−10.68	0.04	0.11
**135**	−10.50	0	0.21	−10.19	0.03	0.31	−10.52	0.06	0.25
**302**	−10.90	0.05	0.55	−10.44	0.05	2.73	−10.55	0.15	0.86
**302a**	−11.17	0.13	0.61	−12.55	0.28	0.56	−10.60	0.08	0.61
**301**	−10.40	0.10	0.92	−10.84	0.07	1.68	−10.85	0.05	0.64
**301a**	−10.66	0.20	0.45	−12.54	0.08	0.86	−11.14	0.15	0.79
MTX	−9.64	0.07	1.87	−9.45	0.15	1.48	−9.54	0.07	1.71

**Table 4:** Binding free energies (kJ/mol) from the MM/PBSA calculations for structure **302** and its derivative **302a** for *L. major* DHFR-TS and *L. braziliensis* DHFR-TS; In both proteins MTX was used as reference ligand.

Structure	Van der Waals (kJ/mol)	Electrostatic (kJ/mol)	Polar Solvation (kJ/mol)	SASA (kJ/mol)	Binding Energy (kJ/mol)
*Leishmania major*					
**302**	−122.6 ± 11.8	−262.3 ± 1.1	263.4 ± 23.5	−16.7 ± 0.7	−138.2 ± 12.2
**302A**	−210.2 ± 10.2	−127.6 ± 3.0	219.5 ± 8.6	−15.9 ± 1.0	−134.2 ± 16.8
MTX	−157.5 ± 12.4	−399.7 ± 10.9	436.4 ± 22.4	−19.4 ± 1.3	−140.1 ± 18.6
*Leishmania braziliensis*					
**302**	−215.4 ± 5.3	−23.6 ± 2.2	124.2 ± 6.8	−20.0 ± 0.4	−134.8 ± 9.5
**302A**	−199.3 ± 6.0	−31.4 ± 0.6	107.4 ± 7.4	−21.3 ± 0.6	−144.5 ± 5.0
MTX	−216.4 ± 5.5	−51.5 ± 3.5	194.6 ± 8.0	−22.6 ± 0.8	−95.9 ± 9.2

## Data Availability

The Supplementary Material can be accesses directly from investigators by email.

## Supplementary Materials

The following supporting information can be downloaded at: https://www.mdpi.com/article/10.3390/antibiotics12040663/s1. Figure S1: Ramachandran plot for hybrid model of, *L. braziliensis*; Figure S2: Ramachandran plot for hybrid model of *L. panamensis*; Figure S3: Ramachandran plot for hybrid model of *L. amazonensis*; Figure S4: Ramachandran plot for hybrid model of *L. major;* Figure S5: Results of a multiple sequence alignment using ClustalW software, comparing the sequences of *Leishmania* and *Homo sapiens*.

## Sample Availability

Samples of all compounds are available from the authors.
