# Supplementary material for: Kaurane-Type Diterpenoids as Potential Inhibitors of Dihydrofolate Reductase-Thymidylate Synthase in New World Leishmania Species
Source: Antibiotics (Basel). 2023 Mar 28;12(4):663. doi: 10.3390/antibiotics12040663 (PMC10135059; doi:10.3390/antibiotics12040663)
Supplement: Supplementary file 1 [file antibiotics-12-00663-s001.zip › antibiotics-2252384-supplementary.pdf]

Hybrid model of *L. braziliensis* DHFR-TS

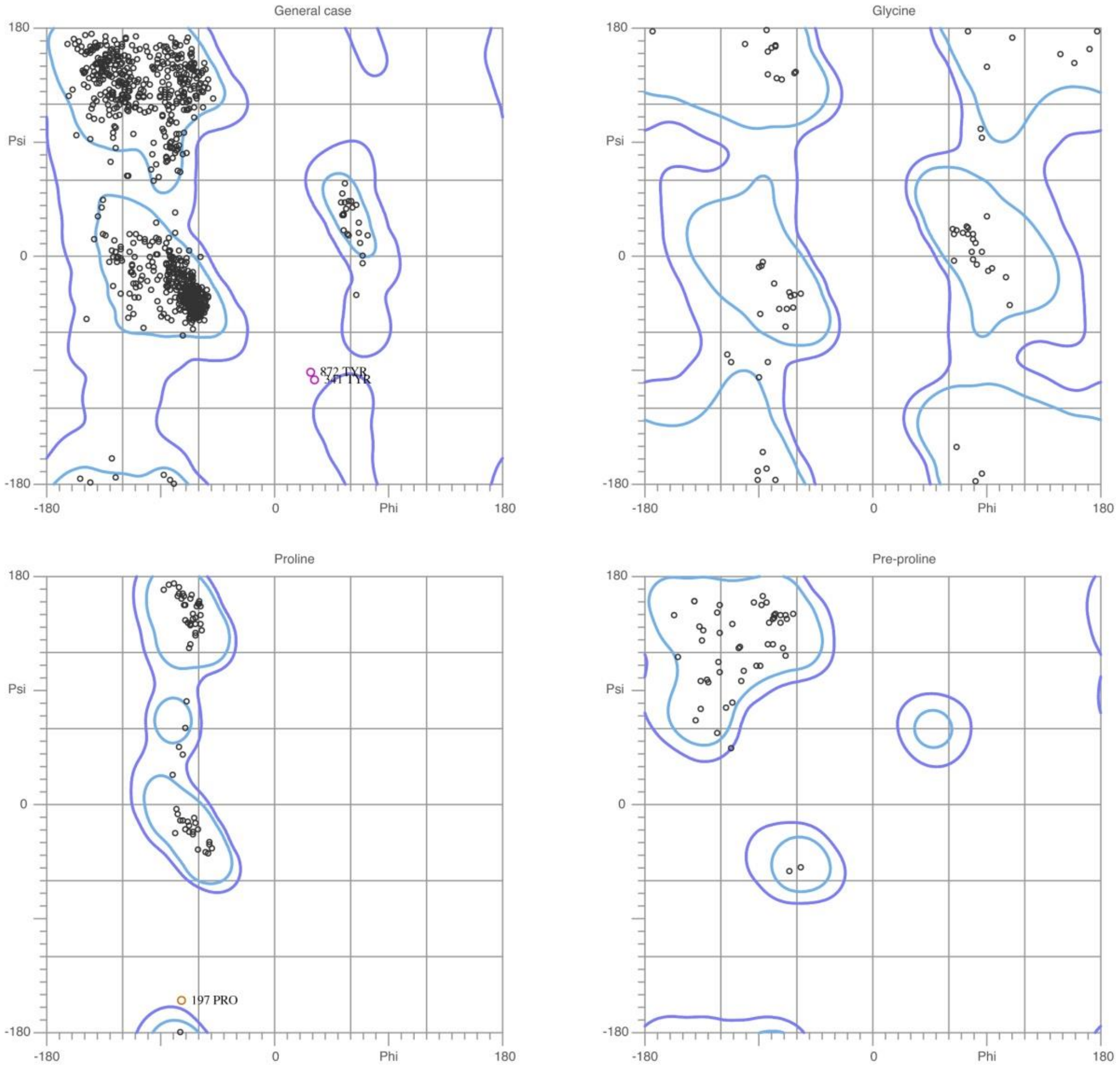

97.3% (1010/1038) of all residues were in favored (98%) regions.
99.7% (1035/1038) of all residues were in allowed (>99.8%) regions.

There were 3 outliers (phi, psi):
197 PRO (-74.1, -154.7)
341 TYR (31.8, -97.6)
872 TYR (28.8, -91.4)

http://kinemage.biochem.duke.edu

Lovell, Davis, et al. Proteins 50:437 (2003)

Figure S1. Ramachandran plot for hybrid model of *L. braziliensis*

Hybrid model of *L. panamensis* DHFR-TS

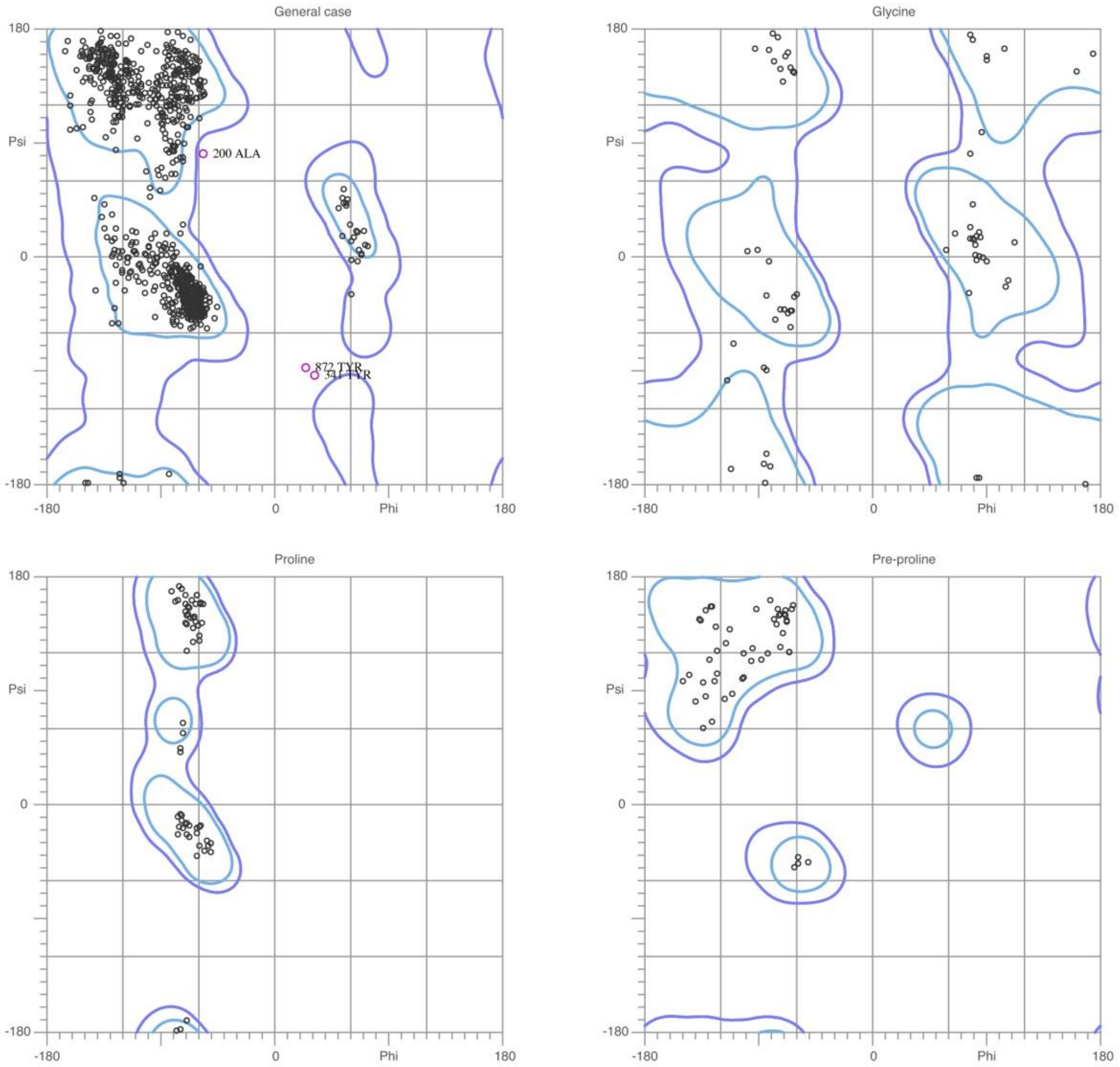

97.2% (1009/1038) of all residues were in favored (98%) regions.
99.7% (1035/1038) of all residues were in allowed (>99.8%) regions.

There were 3 outliers (phi, psi):
- 200 ALA (-57.2, 82.2)
- 341 TYR (31.2, -93.7)
- 872 TYR (24.8, -87.2)

http://kinemage.biochem.duke.edu

Lovell, Davis, et al. Proteins 50:437 (2003)

Figure S2. Ramachandran plot for hybrid model of *L. panamensis*

Hybrid model of *L. amazonensis* DHFR-TS

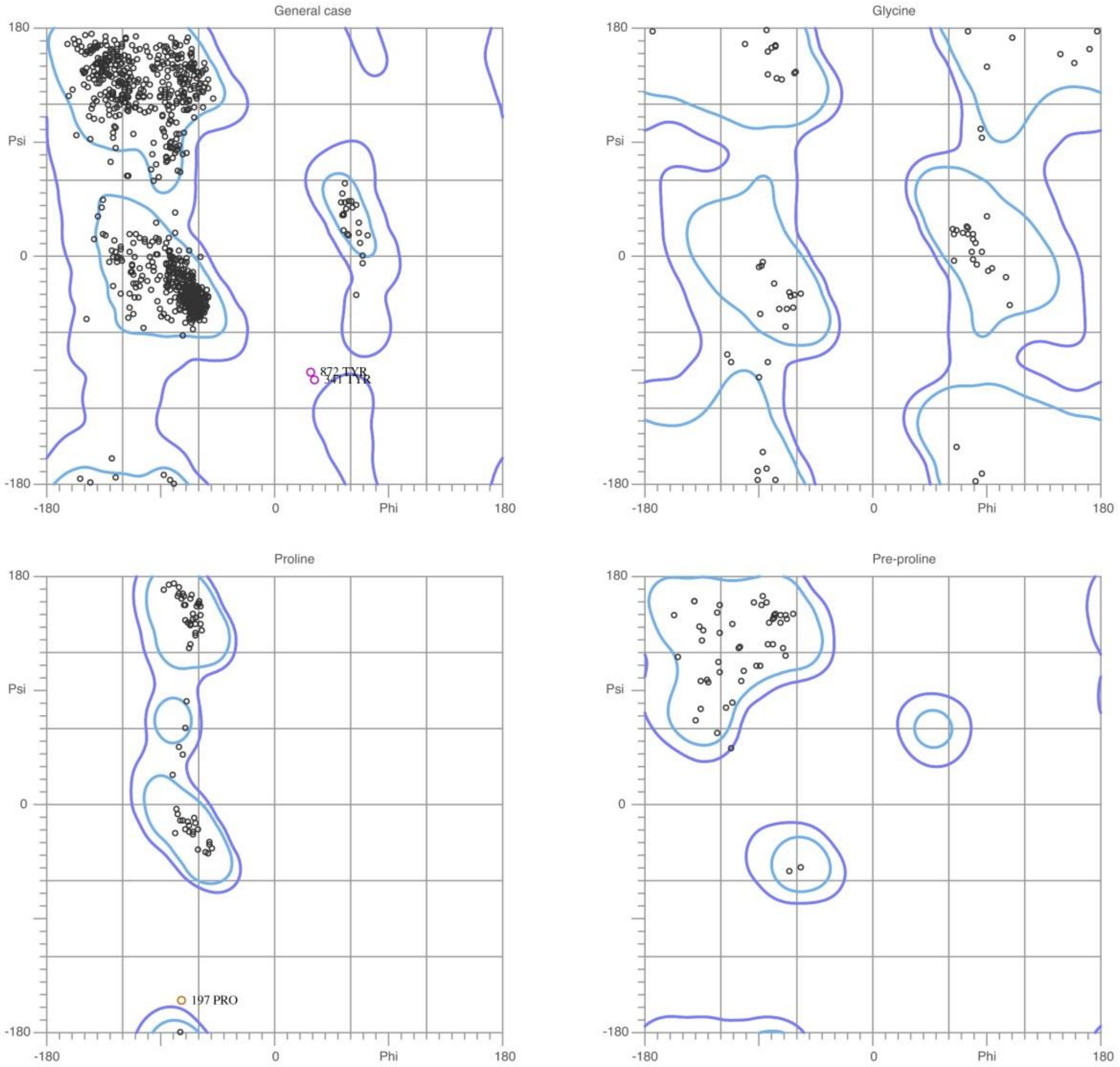

97.3% (1010/1038) of all residues were in favored (98%) regions.
99.7% (1035/1038) of all residues were in allowed (>99.8%) regions.

There were 3 outliers (phi, psi):
- 197 PRO (-74.1, -154.7)
- 341 TYR (31.8, -97.6)
- 872 TYR (28.8, -91.4)

http://kinemage.biochem.duke.edu

Lovell, Davis, et al. Proteins 50:437 (2003)

Figure S3. Ramachandran plot for hybrid model of *L. amazonensis*

Hybrid model of *L. major* DHFR-TS

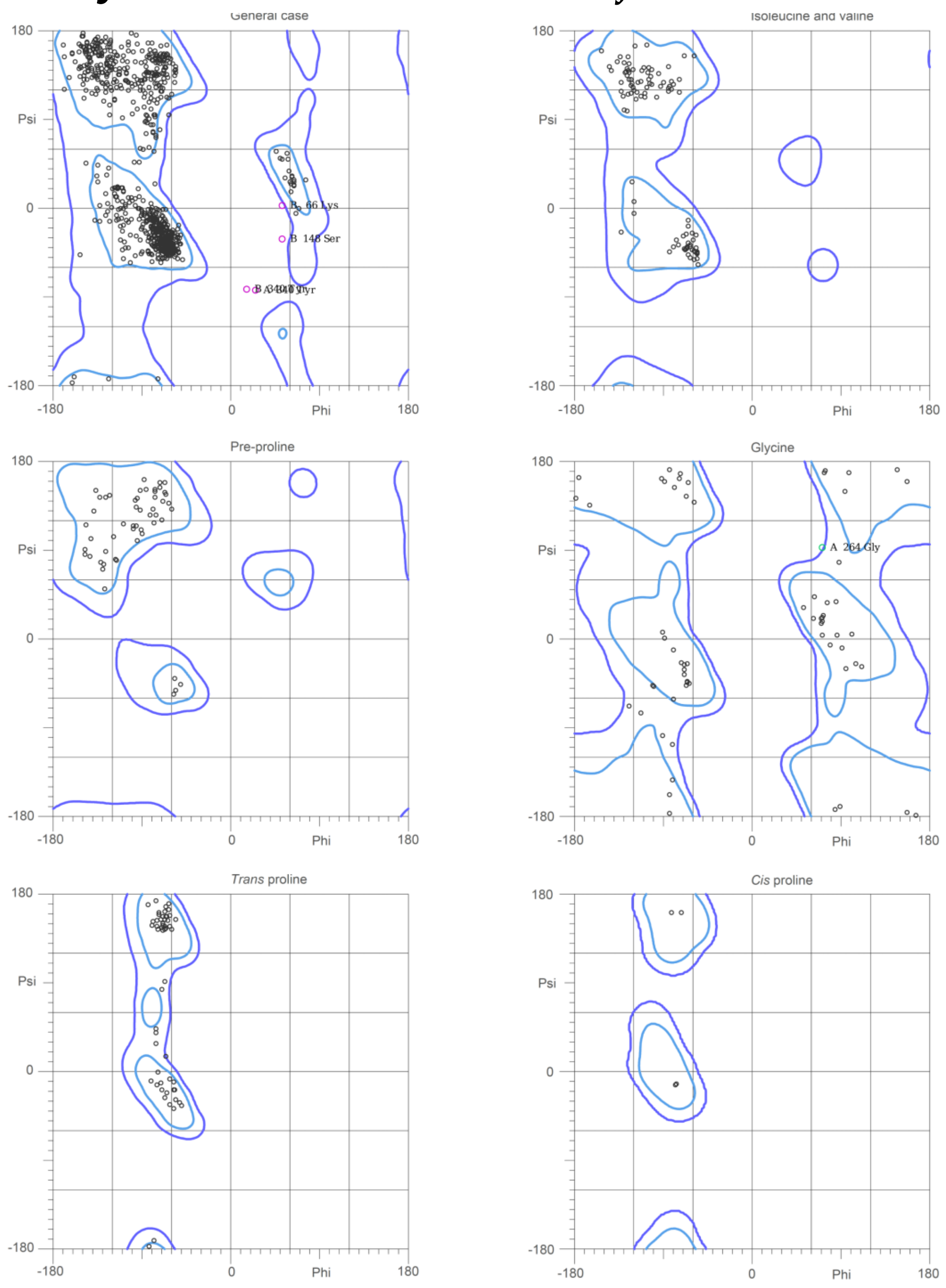

96.9% (1004/1036) of all residues were in favored (98%) regions.
99.5% (1031/1036) of all residues were in allowed (>99.8%) regions.

There were 5 outliers (phi, psi):
- A 264 Gly (72.0, 93.5)
- A 340 Tyr (25.3, -83.2)
- B 66 Lys (52.4, 3.5)
- B 148 Ser (52.5, -31.5)
- B 340 Tyr (16.3, -83.0)

http://kinemage.biochem.duke.edu

Lovell, Davis, et al. Proteins 50:437 (2003)

Figure S4. Ramachandran plot for hybrid model of *L. major*

DHFR Multiple Sequence Alignment

```
Sequence type explicitly set to Protein
Sequence format is Pearson

Sequence 1: DHFR_L.amazonensis    203 aa
Sequence 2: DHFR_L.braziliensis   205 aa
Sequence 3: DHFR_L.panamensis     205 aa
Sequence 4: DHFR_L.major          204 aa
Sequence 5: DHFR_Homo_sapiens     187 aa
Start of Pairwise alignments

Sequences (1:2) Aligned. Score: 78.8177
Sequences (1:3) Aligned. Score: 79.8030
Sequences (1:4) Aligned. Score: 89.1626
Sequences (1:5) Aligned. Score: 20.8556
Sequences (2:3) Aligned. Score: 99.0244
Sequences (2:4) Aligned. Score: 79.9020
Sequences (2:5) Aligned. Score: 24.0642
Sequences (3:4) Aligned. Score: 80.8824
Sequences (3:5) Aligned. Score: 24.0642
Sequences (4:5) Aligned. Score: 23.5294

CLUSTAL 2.1 multiple sequence alignment


DHFR_Lb       ---CFSIIVAVDEQHGIGDGKTIPWQVPEDMAFFKDQTTLLRNKKPPTEKKRNAVVMGRQ
DHFR_Lp       ---CFSIIVAVDEQHGIGDGKTIPWQVPEDMAFFKDQTTLLRNKKPPTEKKRNAVVMGRQ
DHFR_La       ---AFSIVVALDKQHGIGDGESIPWQVPEDMAFFKDQTTLLRNKKPPTDKKRNAVVMGRK
DHFR_Lm       ---AFSIVVALDMQHGIGDGESIPWRVPEDMTFFKNQTTLLRNKKPPTEKKRNAVVMGRK
DHFR_Hs       MVGSLNCIVAVSQNMGIGKNGDLPWPPLRNEFRYFQRMTTTSS----VEGKQNLVIMGKK
                 .:. :**:. : ***..  :**   .:   : :: *   .    .: *:* *:**::

DHFR_Lb       TWESVPLKYRPLKGRLNVVLSSKATVDELLAALPEGKRTAAAQDLVVVNGGLAEALHLLA
DHFR_Lp       TWESVPLKYRPLKGRLNVVLSSKATVDELLAPLPEGKRAAAAQDLVVVNGGLAEALHLLA
DHFR_La       TWESVPVKY-PLKGRLNVVLSSKATVEELLAPLPEEKRAAAAQDIVVVNGGLAAAVRLLA
DHFR_Lm       TWESVPVKFRPLKGRLNIVLSSKATVEELLAPLPEGQRAAAAQDVVVVNGGLAEALRLLA
DHFR_Hs       TWFSIPEKNRPLKGRINLVLS--------------RELKEPPQGAHFLSRSLDDALKLTE
              ** *:* *  *****:*:***               :   ..*.  .:. .*  *::*

DHFR_Lb      RPPHCSSIETAYCVGGAQVYSDAMTAPCVEKLQEVYLTRIYATAPECTRFYPFPPTNAAA
DHFR_Lp      RPPHCSSIETAYCVGGAQVYSDAMTAPCVEKLQEVYLTRIYATAPECTRFYPFPPTNAAA
DHFR_La      RPPYCSSIETAYCVGGAQVYADAMLSPCVEKLQEVYLTRIHTTAPACTRFFPFPPE-NAA
DHFR_Lm      RPLYCSSIETAYCVGGAQVYADAMLSPCIEKLQEVYLTRIYATAPACTRFFPFPPE-NAA
DHFR_Hs      QPELANKVDMVWIVGGSSVYKEAMNHP---GHLKLFVTRIMQDFESDTFFPEIDLEKYKL
             :*  ...:: .: ***:.** :**  *      ::::***       * *  :


DHFR_Lb      AAWDLAWTQGRQRSETGGVEYEIRKYVP
DHFR_Lp      AAWDLAWTQGRQRSETGGVEYEIRKYVP
DHFR_La      TAWDLASSQGRRKSAVDGLEFEICKYVP
DHFR_Lm      TAWDLASSQGRRKSEAEGLEFEICKYVP
DHFR_Hs      LPEYPGVLSDVQEEKGIKYKFEVYEKND
              .   .  .. :..     ::*: :
```

Figure S5: **R**esults of a multiple sequence alignment using ClustalW software, comparing the sequences of *Leishmania* and *Homo sapiens*. Conserved residues are highlighted in yellow, semiconserved residues in gray, and non-conserved residues in red.
